# 

**Published:** 1866-06

**Authors:** 


We are in receipt of the "Descriptive Catalogue of Fluid and Solid Extracts in vacuo; also Concentrations and Officinal Pills." By Henry Thayer & Co., Cambridgeport, Mass.

This is a handsomely gotten up volume of 218 pages, giving, in addition to the formulas for their preparations kept for sale, numerous receipts and formulas, for the benefit of those who like such things.

The Medical Sciences are progressive, and none have made more rapid strides towards perfection than that of Pharmacy. Within the limit of our recollection, Peruvian bark was administered in substance, in the form of extemporaneous mixture of the pulverized bark with water. Not in the form of infusion, but in substance. Now, not only are the active principles separated from the useless and nauseous mass with which they are combined, but enveloped in sugar, so that not the slighest taste is perceived, but of the most pleasant character. Concentrations and Extracts enable the physician to obtain from quantities wonderfully small in bulk, the full effects of remedies once required to be given in large quantities and of such offensive preparations that nothing short of the determined will and vigorous stomach of the patient could accomplish the task. The presence of medicinal agents in the stomach is tolerated, and their effects more certainly secured now than formerly, to say nothing of the unpleasantness to the patient and trouble to the physician.

Contestants for premiums on Essays to be presented to the approaching meeting of the Medical Association of Georgia, are requested to hand in, or forward by mail, their manuscripts to the undersigned, in Atlanta, on or before the 18th of June. Each Essay should be accompanied by a motto, and a separate sealed envelop should inclose the same motto and the name of the writer.

Address A. MEANS, M. D.,
Ch'n Examining Com., Atlanta, Ga.

The catalogue and circular of Albany Medical College is before us. In it we find the names of forty-one graduates for 1865, and a catalogue of all the graduates of the Institution. The next term commences on the first Tuesday in September next; it continues sixteen weeks.

